# Knowledge and Practice Gaps among Pediatric Nurses at a Tertiary Care Hospital Karachi Pakistan

**DOI:** 10.5402/2011/460818

**Published:** 2011-05-03

**Authors:** Rozina Roshan Essani, Tazeen Saeed Ali

**Affiliations:** ^1^School of Nursing, Aga Khan University, Karachi, Pakistan; ^2^Nursing Practice, Division of Nursing Services, Aga Khan University Hospital, Karachi, Pakistan; ^3^Division of Global Health (IHCAR), Department of Public Health Sciences, Karolinska Institutet, Stockholm, Sweden

## Abstract

The advancement in medical science has created health care environments that require nursing professionals who posses specialized clinical knowledge and skills to provide care and deal with critically and acutely ill children. This study explored gaps between knowledge and practice as perceived by the registered nurses of pediatric units by further recommending the changes suggested by them. A descriptive exploratory study design under the quantitative research methodology was utilized using universal sampling of all pediatric nurses working at a tertiary care hospital in Karachi, Pakistan. The gaps between knowledge and practice, as perceived by the participants, were categorized into five major categories: (1) medication (34%), (2) skills (28.3%), (3) knowledge (13.36%), (4) handling of code blue and intubations (12.6%), and (5) operating medical devices (11.58%). As a result, anxiety and incompetency were notably seen in the participants which had great amount of impact on the level of care provided to the patients. The implications of the findings for quality patient care were also analyzed.

## 1. Introduction

Pediatric nurses have challenging role in providing nursing care for the age range from infant till toddler, which requires developmental appropriate care and diligence in assessment of patient and parental concerns [1]. The professional practice of nursing within the pediatric environment can be both rewarding and challenging. Pediatric nurses' activities are complicated and require constant vigilance in providing quality care to the patient. The nurses get limited time to upgrade their knowledge and skills with current advancement in technology. This results in possible gap in the integration of knowledge into practice, whereby they are expected by parents and physicians to be rationalists, knowledgeable, and collaborative on daily basis. 

The literature showing the theory-practice gap in nursing is one of the major challenges, which includes the discrepancy between teaching of theory and clinical practice, when theory should be integrated into practice to reduce the gap in between. Many initiatives have been taken to bridge theory—practice gap; the changes in education are redefining the role of the nurse teachers [2].

Nursing education, both initial and ongoing must be counterpart with contemporary practices. Nursing students should be able to have enough learning experience from both clinical and theoretical perspective. The nurse teacher should have the expertise in theory as well as in practice (Quinn, as cited by [3]). Nurse teachers should have pediatric expertise in theory as well as in practice pertaining to specific skills and knowledge required for providing quality patient care particularly in pediatric settings [4]. 

Further, the literature is consistent in stating that gaps exist between knowledge and practice among both educators and nursing service administrators. This gap should be addressed in order to accomplish quality care by recommending and taking consequent actions for the patient care. 

 This study has allowed the registered nurses, who have undergone 3-4 years of formal nursing education and regular continuing education, to explore and possibly recommend implications that could be implemented to bridge the identified gap between theory and practice and between educators and nurses administrators. As per our knowledge, this study is the first of its kind in Pakistan. The purpose of the study was to identify the knowledge and practice gap and recommendations suggested by participants to improve the quality of care of the pediatric patients.

## 2. Methodology

### 2.1. Setting

The study setting was a tertiary hospital which is located on an 84-acre campus in Karachi, Pakistan. It is comprised of a medical college and a school of nursing, both of which are located at their principal teaching and clinical training site, and its hospital. The hospital has 550 beds in operation, which is equipped to handle medical (including cardiac), surgical, obstetric and, gynecologic, pediatric, and psychiatric patients.

### 2.2. Study Design

This study has been done utilizing the quantitative descriptive study design to identify the knowledge practice gap as perceived by the registered nurses in pediatric units, in Karachi, Pakistan.

### 2.3. Sampling Technique and Sample Size

All nurses involved in pediatric care for more than six months at the time of data gathering in tertiary care hospital were enrolled. This followed the universal sampling strategy. Among total 45 nurses, only 40 who consented to participate in the study were included.

### 2.4. Data Collection

A questionnaire was developed after extensive literature review which went through content and face validity. This validity was done by two faculty members teaching pediatric nursing and one clinical nurse instructor from the hospital setting. Later with the final questionnaire, a pilot testing was also done at Neonatal Intensive Care Unit (NICU). Pilot testing was done to examine the utility of the interview that might be encountered with the use of the tool. Furthermore, it provided a preview of how well the participants understood the issues. After the pilot testing, appropriate revisions on the tool/questioning techniques were done from the similar tertiary care hospital from five nurses. The questionnaire included the variables in five major areas: medications, skills/practice, knowledge, code blue/crash handling, knowledge and practice, and ability to operate complex medical devices. Each area was subdivided in most frequently identified gaps related to knowledge and practice in each category, for example, *medication* included drug calculation, drug dilution, drug compatibility, and pharmacology. *Skills/practice* included pediatric cannulation, catheterizion, nasogastric/oral tube insertion, and suctioning. *Knowledge* included updated knowledge regarding health assessment, disease process, and management and interpretation of lab/diagnostic test results. *Code blue/crash handling* included inadequacy in knowledge and practice in handling intubation and cardiac/respiratory emergencies. *Ability to operate medical devices* included knowledge and practice related to the complex equipment they were using for the care of children, for example, syringe pumps, IVACs, cardiac monitor, and dynamap. 

### 2.5. Ethical Consideration

Permission to conduct this study was obtained from the investigator's thesis committee, the ethical review committee of the university, and the director of nursing services of the hospital. Written informed consent was obtained from the participants, and confidentiality of responses to both the questionnaire and personal data sheet was assured. The participants were provided essential information for informed consent, and their signatures were taken on the consent form. All efforts were carried out to maintain confidentiality and anonymity of the participants. The participants' names were not indicated on the demographic sheets, thus allowing participants to be anonymous. The participants were informed of the right to withdraw from the study at any time and that there would be no potential physical, economical, or legal harm to the participants. All data of the study were kept in locked cabinets. An informed consent was developed in English and was not translated in to Urdu as the participants were all registered nurses and could understand English fully well. It was explained to them that although there would be no personal benefits to the participants, the findings of the study would serve to provide a collective benefit to the nursing profession.

### 2.6. Data Analysis

Data were double-entered and validated before final analysis. The SPSS software (version 14) was used for analyzing the data. Frequencies were calculated for medication, skills/practice, knowledge, code blue/crash handling, and ability to operate medical devices. Descriptive statistics were used to group the data in ways to make it easier to understand. Frequencies and percentages among the knowledge and practice gap identified by the respondents were categorized to identify the most and least frequent.

## 3. Results

Out of total 45 nurses, 40 pediatric nurses consented to participate, showing the response rate of 88.8%. The nurses who participated were mainly from pediatric general wards (60%), followed by emergency and private wings (12.5%), pediatric ICU (10%), and consulting clinic (2.5%) see Table 1. Among the participating nurses, majority (87.5%) were between 20 and 30 years of age. The greater part (85%) was females, and males were 15%. Most of them (72.5%) were single/never married. 55% had diploma in general nursing, 35% had a diploma in nursing as well as in midwifery, and only 10% had, BS degree in nursing. Of the participating nurses, only 55% had their 12-year intermediate, 27.5% did 10 years matriculation, and only 17.5% attained graduate degree in their secular education. Overall, 97.5% were working as full time pediatric nurses and only 2.5% part time. Mean years of experience was 2.6 as pediatric nurse and 2.9 as a nurse. 77.5% of the nurses had experience of only 6 months to 2 years of working as pediatric nurse and 57.5% as nurse. Demographic characteristics of all the study participants are described in Table 2. 

 All the participants perceived gaps in the five major areas and had multiple responses which added up to 449 responses in total; further breakdown of this responses is summarized in Table 3 and narrated below.

### 3.1. Medications

Out of total responses, 153 responses were related to *medications *(*n* = 153, 34%) which were related to medication practice, administration, dilution, dosage calculation, knowledge about the compatibility of drugs, and pharmacology. Almost all the participants verbalized that on day one in pediatric wards, they had heard names of some solutions for the first time as many pediatric calculations like cc/kg/hour were not discussed during their education, which is important with respect to the pediatric population. The participants felt that they were inadequate in this area and did not have any information on the list of intravenous drugs that could be given direct push, the drugs used in emergency situations, dilutions, and compatibility in pediatric patients.

### 3.2. Skills

There were 127 responses related to *skills *(*n* = 127, 28.3%); skills referred to the psychomotor abilities necessary for a nurse to work efficiently and provide high-quality care to the patients. The skills emphasized by the participants as gaps particularly in pediatric setting include cannulation, catheterization, suctioning, nasogastric, and oral gastric tube insertion; they were not aware about the sizes of BP cuffs, catheters, nasogastric tubes used for different age/weight, and developmental stagewise. They shared that although they were comfortable doing neurological assessment in adults they were not able to do so when it comes to children. As a result, they have to use trial and error in procuring the cannulas and catheters for use and thus were not able to provide quality care to their patients.

### 3.3. Knowledge

There were 60 responses related to *knowledge *(*n* = 60, 13.36%)*; *these included knowledge about health assessment, disease process, signs and symptoms, management and interpretation of lab results, the diagnostic test results, and preoperative and postoperative care/education particularly in pediatric setting. They found it difficult to relate the signs and symptoms with the diagnosis for pediatric-related diseases and conditions. They shared that the pediatric rotations were only for 6 weeks and thus care of pediatric patients were not adequately covered in the course content; hence, caring for these patients in actual practice was difficult for them. They felt that due to this they were not able to explain/provide teaching to the child and parents which created anxieties among the graduates.

### 3.4. Crash/Code Blue Handling and Intubation

There were 57 responses related to *crash/code blue handling and intubation *(*n* = 57, 12.6%). The respondents verbalized that they could not handle cardiac arrest and felt that they had inadequate knowledge and skills to handle any code blue/intubation. They shared that they are never taught how to secure endotracheal tube when the intubation is done by the anesthesiologist. Thus they never came forward in emergency situation and forwarded the task to their seniors, and they eventually are not able to gain any exposure for this type of scenario. In a way, they hide their inadequacies and are not able to provide quality care to their patients.

### 3.5. Inability to Operate Medical Devices

There were 52 responses related to *inability to operate medical devices *(*n* = 52, 11.58%) during the professional job life, which included operating advanced technologies at the bedside which are very important for the care of patients. Among the commonly used machinery at the patients' bedside which nurses felt uncomfortable using or manipulating particularly in the pediatric units were cardiac monitor, defibrillator, phototherapy, incubator, space lab, ECG machine, syringe pump, IVAC, and IMED which regulate the flow rate of the drugs. This makes nurses feel helpless and incompetent in front of the patients and the family members and thus compromise quality patient care.

## 4. Discussion

The study discovered five major gaps among the pediatric nurses, which were related to medications, skills, knowledge handling emergency situations, that is, crash/code blue handling, and operating medical devices. Gaps related to medication remain the most frequently identified gaps; our study participants were younger (mean age 25.9 years) and had experience as pediatric nurse of 6 months to 2 years, which could be the reason that medications category came out to be the major gap because dosages, and dilutions as a course taken in basic nursing education only carries 4 credit hours for pharmacology and only 2 credits for mathematics throughout the 3-year diploma program and the 4 year-BSN program. Nobody can deny the fact that understanding of pharmacology and its application to patient care is an integral part of pre-and postregistration nursing education, with the current trend towards extending nurse prescribing. [5]. Nurse's roles which were identified as requiring pharmacology knowledge included drug administration, patient assessment, prescribing by nurses, and patient medication education. King's [6] study findings revealed a limited understanding of the subject and dissatisfaction with the teaching method of pharmacology. The authors have suggested that there is a theory-practice gap in this area of curriculum. Literature [7] revealed higher proportion of medication errors in pediatric (67%) as compared to adult nurses (56%). The medication error rates per 1,000 patient-days computed from actual occurrence reports were also higher in pediatrics as compared to adult units. Prot et al.'s [8] study in a pediatric teaching hospital reported (27%) errors related to timings, routes, dosages, unordered drugs, and so forth. 

There are some standard skills of knowledge and attitudes that are expected from the pediatric nurses to provide high-quality care to the patients and their families. As a pediatric nurse, there is a wide range of knowledge required to cover child care specialty. Pediatric nurses are now expected to possess skills as well as knowledge in areas related to child development and understanding of disease processes. The lack of information may as a result produce stress in nurses and thus impact on the quality of care in a variety of ways like feelings of depression, psychological distress, burden of not knowing things, and feelings of being pressurized. A study done by Hemani [9] in Pakistan emphasized that due to nurse's lack of technical and professional knowledge, doctors treated them as their handmaidens rather than as their colleagues. She professed that the only answer to this was self-improvement which would bring self-respect and self-confidence and enable the nurses to prove their worth.

 The result of this study also highlighted that there were also gaps related to psychomotor skills, clinical knowledge that nurses perform in pediatric settings including the skills which are very crucial for patient care. Suleiman [10] highlights that nurses stated that very little time is given to the student nurse for their clinical practice in their student life to provide quality care to patients when they step in their professional life. A study concluded [11] that nursing students must be adequately prepared to carry out clinical skills competently and efficiently and educators and practitioners must display the knowledge and skills required to promote theory-practice integration to enhance nursing education, which in turn will optimize high standards of patient care. The participants also recommended increasing the number of clinical hours to sharpen and brighten their hands on skills specifically related to pediatric population. Martin and Mitchell [12] asserted that the level of clinical exposure afforded to nursing students during rotations is considered typically limited by the participants because of which they lack clinical expertise. They recommend that it is necessary to supplement curriculum with additional clinical experience. They feel that credit hours should be increased and the curriculum should be more focused for both the adult and the pediatric components as it is not enough. Literature confirms the importance of pediatric clinical skills in the practice arena. It is essential, therefore, that pediatric nurses be proficient in performing the vital skills necessary for care of children. 

The results also indicated that pediatric nurses highlighted gaps related to knowledge; however, in today's challenging world, the nurses, specifically the pediatric nurses, active participation in planning the care of patient is vital. [13]. Nursing education today must prepare nurses for the future, as health care is dynamic, nonstatic, and constantly in a state of flux. There is a great need for both strong theoretical background and an equal amount of hands-on clinical experience as it is necessary to expand the view of a nurse's role [14]. This is only possible when the nurses are knowledgeable and well informed. The curricula of today must prepare mentors of the future by instilling in them the type of knowledge and skills which will be required over the course of their careers [15]. 

 Nurses are generally the first responders to a cardiac arrest and initiate basic life support while waiting for the advanced cardiac life support team to arrive. Speed and competence of the first responder are factors contributing to the initial survival of a person following a cardiac arrest. Hence, knowledge, skills, and attitudes of individual nurses may influence the speed and level of involvement in true emergency situations. Resuscitation knowledge and skills are important for the chosen career, and the current pediatric nurses curricula may not be providing sufficient experience to develop adequate skills, fund of knowledge, or confidence needed for resuscitation. Participants found that training related to CPR was insufficient. Nurses' ability to assist in emergencies involving Advance Cardiac Life Support (ACLS) showed that they had inadequacies in their performance. The findings in the study are similar to those in other countries with regard to abilities in handling ACLS cases. 

While effective acquisition of technical skills is essential for quality pediatric care, little is known about how technical skills are learned in the pediatric setting. The evolution of biomedical technology has led to an extraordinary use of medical devices in health care delivery. Recently, much emphasis has been given to patient safety, and, thus, it is crucial that the health care professional specialists involved in direct patient care should be well aware and competent enough to deal with the medical devices available in the units where they work. 

The technological, scientific, and medical advances have created neonatal and pediatric health care environments that require nursing professionals who possess not only highly specialized clinical knowledge and skills to provide care but also the technical expertise to manage sophisticated medical technology [16]. In this study, the nurses' feelings of inadequacy about the use of sophisticated technology could have been augmented by their own initiative to be active learners rather than passive receivers of knowledge. This attitude could have been instilled in them during their formal education both in the school of nursing and the hospital. 

 The above discussion with lines of evidence from the literature shows that the studies done worldwide were consistent with the finding of this study. It further shows that problems encountered by health care personnel, specifically nurses, are similar and not conflicting with findings in other countries. However, it appears that more problems exist in the local pediatric setting, emphasizing the need to bridge knowledge and practice gap. The participants also shared their feelings of inadequacies, insecurities, and anxieties when caring for patients in the pediatric units, see Table 4. They also added that due to this feeling, they are unable to provide quality care to patients. 

The *Recommendations* were made by participants (see Table 5). They made recommendations with respect to either nursing curricula or in-service education. There was an agreement among participants that the pediatric nursing course, including its clinical component, was not enough to prepare them for their future practice as a competent nurse. There was a consensus that in-service education should reinforce knowledge and skills learned from nursing education, and ongoing updates on current trends and practices in pediatric nursing should be a part of the nursing education programs, both centrally and at the unit levels. 

The recommendations from this study are addressed to nursing services, nursing education, the newly hired nurses, and future research. For nursing services, it is recommended that the Nursing Education Services (NES), which is responsible for in-service training, reevaluates their approaches for education to meet the increasing needs of new graduates due to the fast changing technological advancement. The current setup does not fully help the newly hired nurses, as expressed by the respondents. Senior nurses should play a vital role in staff education as mentors and role models and should be given incentives for these roles. Current training programs, although in place, may not be providing enough experience to develop adequate skills, knowledge, or self-confidence, needed for a pediatric nurse to work effectively in this specialty, and to provide high quality care to the patients. Fast tract refresher courses and workshops should be conducted more frequently within the institution, as part of the continuing education program. 

For nursing education, including nursing curriculum, it is recommended that clinical hours in the curriculum at the school of nursing should be increased. To enhance learning during this period, final year summer clinic should be planned in the same area where the students will most probably be assigned to their professional life after graduation. There should be collaboration between the school of nursing and the nursing services to develop curriculum courses, teaching more reality-based clinical aspects and components that could be easily applicable and integrated by the nurses while in the actual practice setting. Nursing educators must constantly monitor clinical practice and re-evaluate the curriculum to ensure that necessary knowledge and skills for successful practice are achieved from the educational program. Ferguson and Jinks [17], identified factors that need to be considered in addressing theory-practice gaps, which include organization and sequencing of theory and practice in the curriculum, the role of tutors in the clinical area, the teaching responsibilities of clinical staff, the move to higher education, the influence of the hidden curriculum, and the growth of nursing theories and research.

For further research, it is recommended that a study of similar nature be done in other specialties, hospitals, and provinces of the country. Qualitative research could be conducted on the same issue so that the perceptions of the nurses on the knowledge-practice gaps could be explored in-depth. Lastly, it is recommended that nursing students and new graduates who enter the practice setting take responsibilities as adult learners. They should have the initiative to learn things on their own, using various resources available, especially when this learning will help them improve their practice by adding to their level of confidence.

The *study limitations* were related to time and convincing nurses to participate, as the study was a part of MSN program and had to be completed within a given time frame. Securing consent and time from the research participants was tedious and challenging as the respondents had reservations with regard to answering some questions which they considered threatening to their jobs and positions, since this was the first time a study has been carried out in this area. However, these factors were minimized by providing detailed explanations about the study purpose, maintaining an environment conducive for the interview, and promising anonymity and confidentiality.

## 5. Conclusion

The findings of this study provided the support needed for the observed gaps in knowledge and practice among nurses, through scientific research. The gaps identified by the respondents were based on their own experiences, as they experience them in their professional practice. It provided essential information on areas in nursing education and nursing practice which need to be improved as initially there was little awareness about the magnitude of these gaps. Furthermore, the study enabled the researcher to recognize that the attitude for lifelong learning, as presented by the respondents, is limited and needs to be enhanced. If quality of patient care remains to be the ethos of nursing, then these gaps need to be bridged with both nursing education and nursing services working in unison.

## Figures and Tables

**Table 1:** Number of registered nurses selected from the different wards (*n* = 40).

Sr. no	Units	Number of RNs in each ward	% Taken from each ward	Number of sample from each ward	% of participants selected from each ward
(1)	Pediatrics A	21	90.4	19	47.5
(2)	Pediatrics B	05	100	05	12.5
(3)	Pediatric ICU	12	33.3	04	10
(4)	Emergency room	30	16.6	05	12.5
(5)	Private wing II	18	27.7	05	12.5
(6)	Pediatric consulting clinic	1	100	1	2.5
(7)	Community health centre	1	100	1	2.5

	Total	88	45.4	40	100

**Table 2:** Demographic description of samples (*n* = 40).

	Frequency	%
Age		

20–30 years	35	87.5
31–40 years	04	10
40 and above	01	2.5
*Mean age 25.9 years*		

Gender		

Female	34	85
Male	06	15

Years of graduation		

1986–1990	01	2.5
1990–1995	02	5
1996–2000	03	7.5
2001–2005	34	85

Years of experience as nurse		

6 months–2 years	23	57.5
2–4 years	12	30
4–6 years	02	5
6–8 years	00	0
8–10 years	03	7.5
*Mean years of experience 2.9 years*		

Years of experience as pediatric nurse		

6 months–2 year	31	77.5
2–4 years	03	7.5
4–6 years	04	10
6–8 years	00	00
8–10 years	02	5
*Mean years of experience 2. 6 years*		

Marital status		

Married	11	27.5
Single	29	72.5

Organizational graduated from		

AKUSON	28	70
Non-AKUSON	12	30

Professional qualification		

Diploma in nursing	22	55
BSN	04	10
Diploma in nursing and midwifery	14	35

Academic qualification		

Matriculate	11	27.5
Intermediate	22	55
Graduation	07	17.5

Employment		

Full time	39	97.5
Part time	01	2.5

**Table 3:** Total reponses = 449.

Major gaps	No. of Reponses	Subcategory %	Overall category %
*(I) Medications *	153		153/449 = 34%

(i) Dosage calculation		50/153 (32.68%)	
(ii) Drug dilutions		40/153 (26%)	
(iii) Pharmacology		40/153 (26%)	
(iv) Compatibility of drugs		23/153 (15%)	

*(II) Skills*	127		127/449 = 28.3%

(i) Pediatrics-specific skills, for example, cannulation, catheterization, nasogastric/oral tube insertion, and suctioning		58/127 (45.67%)	
(ii) Unaware about different sizes according to age of children		42/127 (33.07%)	
(iii) Feeling incompetent and not confident to perform pediatric skills		27/127 (21.26%)	

*(III) Knowledge*	60		60/449 = 13.36%

(i) Health assessment		09/60 (15%)	
(ii) Disease process/diagnosis		19/60 (31.67%)	
(iii) Management of pediatric patient		25/600 (41.67%)	
(iv) Interpretation of lab/diagnostic test results		07/60 (11.67%)	

*(IV) Code blue handling or intubation *	57		57/449 = 12.6%

(i) Inadequacy in knowledge and practice in handling pediatric intubation		24/57 (42.11%)	
(ii) Inadequacy in knowledge and practice in handling pediatric cardiac/respiratory emergencies		33/57 (57.89%)	

*(V) Operating medical devices/equipments*	52		11.58%

(i) Knowledge and practice related to complex medical equipments/devices, for example, syringe pumps, IVACs, cardiac monitor, and dynamap		52/52 (100%)	

*Total*	449		

**Table 4:** Effects on quality care/personal feelings of participants due to these gaps*.

	Feelings identified by the participants	Responses
(1)	We feel embarrassed when we do not know how to handle the pediatric population in front of parents.	15
(2)	The parents, during hospitalization, expect too much from us, which makes us feel incompetent in front of them, and if we fail to answer or satisfy them, they complain.	19
(3)	Child and parents both are anxious because of a new nurse assigned to them, because we lack confidence.	12
(4)	We feel incompetent to teach to the parents because of these gaps, and, thus, quality care is compromised.	20
(5)	When we don't know any skills or when we lack knowledge on rounds, our image becomes low and thus the doctors also complain for it.	09

	Total	75

*Directly quoted from the participants.

**Table 5:** Recommendations to bridge knowledge practice gaps as verbalized by the participants.

Recommendations for:	Recommendations as verbalized by the participants*
Nursing education	(i) Credit hours for both pediatric theory and practice should be increased. (ii) The faculty should make the students more independent on clinicals. (iii) The students should be given responsibilities of the patients assigned wholly and solely and they should hand “over” to the upcoming shift staff. (iv) Written work assignments during the clinical on wards/units should be decreased so that students take more interest in what is going on with the patient and take active part in teaching and procedures. (v) The clinical hours should be until 3 PM instead of 1 PM, so that the students do not run away without doing/completing their responsibilities. (vi) The faculty should not discuss the patient in isolation in a room, but at the bedside to double check it with the staff on duty, and also to facilitate more learning about the assigned patients. (vii) There should be pediatric specialized courses for at least 6 months, which could provide nurses with special skills and knowledge for caring effectively. (viii) More practice for medication calculation should be encouraged through live scenarios. This could be integrated within each specialized course taught throughout the curriculum and not as a separate course in isolation. (ix) Crash cart handling should be introduced in nursing education. (x) Clinicals should also be planned in the evening and night shift at least once a week during the clinical week with the faculty. (xi) Summer clinicals should be preplanned such that each and every student should get exposure to each specialty in the hospital, during their 3/4 year of formal studies. It should be a trio model and the students should not be handed over to the head nurses for scheduling on their own and using them as nursing assistant.

Nursing services In-service training programs	(i) The orientation of NES should not be adult focused but it should also cover the pediatric population in all aspects of teaching. (ii) The medication recertification should also focus on pediatric dosages and scenarios. (iii) The cannulation should focus on both peads and adults. (iv) On joining the orientation program the NES instructor should know in which unit the RN will be assigned so that they can focus on the identified areas. (v) Modules should be prepared for all specialties, whether the person is going to the peads, or critical care, or surgery, and so forth. Thus orientation should be given to subgroups of specialty after need assessment. (vi) NES instructors should also be available for at least the initial months on shifts so that if the staff nurses are busy they can come to help or guide the novice nurses in the unit. (vii) The NES should cover the drug dilution file, which contains the drugs and dilution, so that emergency drugs are used during the cardiopulmonary arrest. They should take a quiz on it before sending the nurse to the unit. It should contain both peads and adult scenarios. (viii) NES library should have enough recent books and journals according to the specialty, the issuing systems should be user friendly, and the book should be issued at least for 3 days. (ix) During the orientation days, the management should provide a list of options of placements to the NES instructor. The orientees should be given at least three options to select and finally leave it to the group to decide among the available vacancies in the hospital units. This will allow the orientees to make decision and feel important that they have decided their placements according to their previous clinical exposures and eventually feel good throughout their professional life.

Clinical nurse instructors in the hospital settings	(i) The clinical nurse instructor (CNI) should focus on the case studies method, which will enhance critical thinking in the staff, and they will question more and learn more. (ii) There should be recent materials like books and journals available in the wards so that they could be utilized as references when needed during free time, to clarify some day to day issues. (iii) The intranet should have enough education materials for ongoing learning. (iv) The on-the-job training course for 1 week could also serve the purpose, if offered during the first month of joining as a staff nurse. (v) The CNIs should report to a separate clinical manager who reports to the director. The CNIs should constantly plan continuing education for the nurses on the ward, not only when novice nurses come on the ward. They should not be involved in management work; this should be done only by the head nurse.

*Directly translated to English from Urdu or directly quoted from the participaants.
